# *In Vitro* and *In Silico* Vibrational–Rotational Spectroscopic Characterization of the Next-Generation Refrigerant HFO-1123

**DOI:** 10.1021/acs.jpca.2c04680

**Published:** 2022-08-05

**Authors:** Nicola Tasinato, Andrea Pietropolli Charmet, Giorgia Ceselin, Zoi Salta, Paolo Stoppa

**Affiliations:** † Scuola Normale Superiore, SMART Laboratory, Piazza dei Cavalieri 7, I-56126 Pisa, Italy; ‡ Dipartimento di Scienze Molecolari e Nanosistemi, Università Ca’ Foscari Venezia, Via Torino 155, I-30172 Mestre, Italy

## Abstract

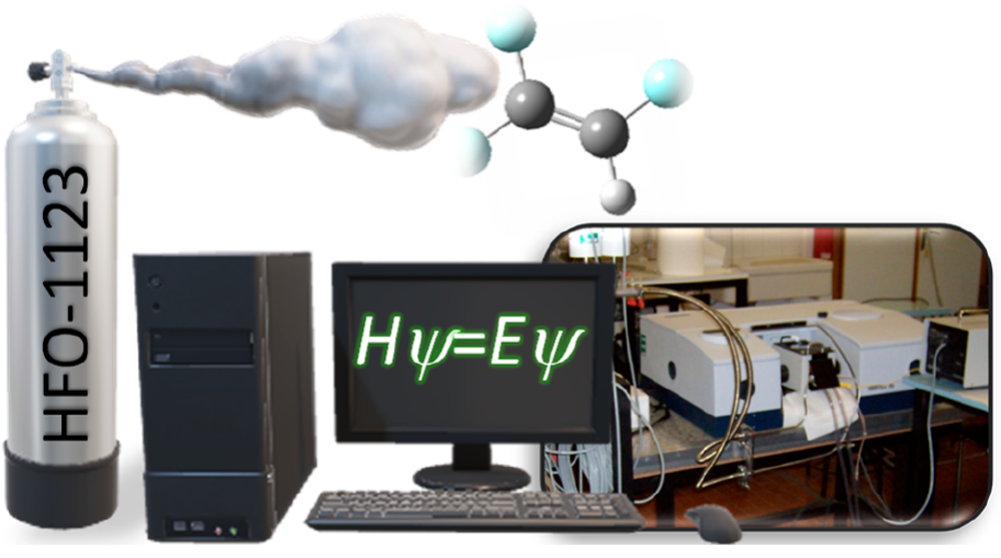

Very short-lived substances have recently been proposed as replacements for hydrofluorocarbons (HFCs), in turn being used in place of ozone-depleting substances, in refrigerant applications. In this respect, hydro-fluoro-olefins (HFOs) are attracting particular interest because, due to their reduced global warming potential, they are supposed to be environmentally friendlier. Notwithstanding this feature, they represent a new class of compounds whose spectroscopic properties and reactivity need to be characterized to allow their atmospheric monitoring and to understand their environmental fate. In the present work, the structural, vibrational, and ro-vibrational properties of trifluorothene (HFO-1123, F_2_C = CHF) are studied by state-of-the-art quantum chemical calculations. The equilibrium molecular structure has an expected error within 2 mÅ and 0.2° for bond lengths and angles, respectively. This represents the first step toward the computation of highly accurate rotational constants for both the ground and first excited fundamental vibrational levels, which reproduce the available experimental data well within 0.1%. Centrifugal distortion parameters and vibrational–rotational coupling terms are computed as well and used to solve some conflicting experimental results. Simulation of the vibrational transition frequencies and intensities beyond the double harmonic approximation and up to three quanta of vibrational excitation provides insights into the couplings ruling the vibrational dynamics and guides the characterization of the gas-phase infrared spectrum experimentally recorded in the range of 200–5000 cm^–1^. The full characterization of the IR features is completed with the experimental determination of the absorption cross sections over the 400–5000 cm^–1^ region from which the radiative forcing and global warming potential of HFO-1123 are derived.

## 1 Introduction

Halogenated organic molecules, when released into the environment, influence atmospheric chemistry and, hence, Earth’s climate by contributing to either ozone depletion or global warming. Due to this role as trace gas pollutants, over the years, halocarbons have been the subject of many experimental and theoretical investigations aimed on the one side at allowing their monitoring and on the other at understanding their atmospheric reactivity.^1−3^ Halogenated hydrocarbons are in general widely used as blowing agents, propellants, refrigerants, and fire extinguishers, and halogenated olefins are also widely employed to produce synthetic polymers and copolymers characterized by a good mechanical, thermal, and chemical resistance. However, during the last three decades, a transition from chlorofluorocarbons (CFCs) to hydro-chlorofluorocarbons (HCFCs) and then to hydrofluorocarbons (HFCs) has taken place, driven by the need of mitigating stratospheric ozone depletion. In fact, the Montreal protocol that was enforced in 1987 has been phased out with a few exceptions: the use of CFCs since 1996 due to their capacity to catalytically destroy stratospheric ozone molecules. This feature comes from the chlorine atom that, once in the stratosphere, acts as a catalyst in the ozone depleting reaction cycles. In addition, CFCs are also greenhouse gases because they strongly absorb the electromagnetic radiation within the atmospheric window located in the 8–12 μm wavelength spectral region. Indeed, these molecules generally show very strong infrared (IR) absorption bands due to C–F and C–Cl stretching vibrations. To make things worse, CFCs generally have long atmospheric lifetimes that on the one side favor their accumulation in the atmosphere and on the other side contribute even more to their global warming activity in the long period. As replacement gases, HCFCs and HFCs with analogous chemical and physical properties have been proposed. Between the two, HFCs have appeared to be the more convenient option because, given the absence of chlorine atoms, they cannot contribute to ozone loss. Furthermore, these molecules present shorter atmospheric lifetimes than CFCs, because the C–H bond undergoes a reaction toward hydroxyl radicals in the troposphere.^4^ Nevertheless, these gases still contribute to global climate changes as the presence of the C–F chromophore makes them greenhouse gases that absorb IR radiation around 9 μm.

In 2016, the Kigali amendment to the Montreal protocol posed the onset of a new transition with the requirement to reduce HFCs emissions and to switch to the use of compounds characterized by a low global warming potential (GWP). Low-GWP molecules are currently being developed in order to meet the new regulation, and hydro-fluoro-olefins (HFOs) have appeared to be a good solution for applications in the field of refrigeration and air conditioning. In 2014, Asahi Glass Co., Ltd. employed trifluoroethene (F_2_C=CHF), known as HFO-1123, as the main component of refrigerant blends characterized by extremely low GWPs. As such, HFO-1123 is emerging as a new molecule in the refrigeration market, and it is expected to be a next-generation refrigerant for air conditioners.^5^ From a chemical point of view, presenting unsaturated C=C bonds, HFOs have low chemical stabilities with a consequent shortening of the atmospheric lifetime that is, in turn, at the basis of the low GWP. This increased chemical reactivity however may cause self-decomposition reactions, and in fact, it has been reported that HFO-1123 undergoes a disproportionation reaction under high-temperature and high-pressure conditions.^6,7^ As such, the thermodynamic properties of this molecule as well as its binary mixtures with other fluorinated refrigerants have been studied in recent years (see refs (8−11) and the references therein). Furthermore, HFO-1123 reactivity toward the OH radical has also been investigated.^12^

Among the different techniques available for environmental and process monitoring, one of the most effective and easy-to-use is IR spectroscopy whose successful application, however, depends on the availability of accurate spectroscopic data, above all the transition frequencies and intensities, for the species of interest.^1,13,14^ While high-resolution spectroscopic investigations can be exploited to obtain the requested information to a very high accuracy, the necessary ro-vibrational analyses are complicated by the presence of resonances and very often by the spectral congestion due to the presence of low-lying vibrational states.^15,16^ The determination of line intensities requires a careful line-by-line approach^17,18^ that, being hampered by the overlap among different spectral features, can often be effectively exploited only for the lighter species. Vibrational assignments and band intensities, besides delivering the fundamental knowledge that is instrumental for the ro-vibrational analysis of high-resolution IR spectra, provide the description of vibrational couplings occurring within the molecule.^19−21^ This data, coupled with sophisticated experimental methods capable of simplifying the spectral structure (like cooling cells^22,23^ and supersonic jet expansion^24,25^) and with purposely tailored data-analysis software,^26−28^ aids and speeds up the huge and time-consuming task of interpreting high-resolution spectra.

On their own, vibrational investigations are ever increasingly supported by quantum chemical (QC) calculations that work as a guide and a support for the assignment of the experimental signals. In fact, reliable predictions of vibrational transition frequencies and IR intensities can be obtained beyond the rigid-rotor-harmonic-oscillator model without the need of any empirical scaling factor and with a full account of anharmonic resonances with accuracies within 10 cm^–1^ for fundamental frequencies and a few km mol^–1^ for IR intensities.^29,30^ Concerning halocarbons, it has been demonstrated that such accuracy can be reached using the coupled cluster theory with singles, doubles, and a perturbative estimate of the triples, CCSD(T),^31,32^ coupled to large basis sets.^33−35^ An alternative and more cost-effective approach is represented by QM/QM' computational protocols involving CCSD(T)-based composite schemes for geometry and harmonic properties,^36^ coupled with anharmonic effects described through density functional theory (DFT).^37,38^

The microwave spectra of HFO-1123 were investigated about 50 years ago, leading to the determination of the ground and some excited vibrational level rotational constants and to an effective structure,^39^ which was later refined using microwave and electron diffraction data.^40^ The rotational spectra were reanalyzed by obtaining centrifugal distortion terms some years later,^41^ while more recently, improved rotational parameters have been determined for the ground and some low lying vibrational excited states together with the analysis of the ro-vibrational spectrum of the ν_6_ band.^42^ The vibrational spectrum of trifluoroethene was measured a long time ago,^43^ while in the middle of the 70s, it was considered in a study devoted to explore the C–H stretching frequencies in a series of halogenated ethenes.^44^ Some high-resolution investigations were carried out in more recent times with the aim of determining the molecular parameters in some excited vibrational levels, even though the observed irregularities in the spectra patterns, due to Coriolis and anharmonic interactions, were only recognized without any formal treatment of the couplings.^45−47^ The vibrational properties of HFO-1123 were the subject of theoretical computations within the harmonic approximation,^48,49^ and more recently, Jiang et al. compared the available experimental data on fundamental frequencies with the corresponding values computed by scaled ab initio calculations using the B3PW91 functional.^50^

As the characterization of the vibrational spectrum of trifluoroethene dates back to almost 70 years ago and an accurate modeling of its ro-vibrational spectroscopic properties has never been undertaken, the present work deals with a combined experimental and theoretical study of the vibrational and ro-vibrational properties of HFO-1123 in order to fill these gaps in view of the renewed interest in this species for applications as a refrigerant of the next generation. Experimentally, the vibrational quantum assignment of the gas-phase IR spectrum is carried out between 200 and 5000 cm^–1^ and the determination of integrated absorption cross sections is performed over the 400–5000 cm^–1^ range. The vibrational analysis is guided by state-of-the-art quantum chemical calculations involving hybrid CCSD(T)-based composite schemes and DFT anharmonic calculations. These are combined into hybrid force fields from which vibrational energies, transition strengths, rotational parameters, and coupling constants are determined.

## 2 Experimental Details

To record the gas-phase IR spectra, two different Fourier-Transform IR (FTIR) spectrometers were used according to the spectral region. In the range between 200 and 400 cm^–1^, the spectra were recorded at a resolution of 1.0 and 0.5 cm^–1^ using a Spectrum One (PerkinElmer) interferometer and a glass cell (optical path of 15 cm) equipped with KRS-5 windows. In the range of 400–5000 cm^–1^, the spectra were measured by a Bruker Vertex 70 spectrometer with a resolution ranging from 1.0 to 0.2 cm^–1^ and using a double-walled, stainless-steel cell (optical path of 13.4 cm) equipped with KBr windows. Cross section measurements were performed at a resolution of 0.5 cm^–1^, keeping the cell temperature constant at 293.0 K (±0.5 K). The pressure of the sample was varied in the range of 1.1–222.5 hPa, and for each experimental run, 128 interferograms were collected to maximize the signal-to-noise ratio. Following the methodologies established in previous works,^33,51^ distortions due to finite-resolution effects^52^ were minimized by mixing the sample with N_2_ (SIAD, purity >99%) up to a total pressure of 101 kPa. Adsorption of the sample on the cell walls, checked by monitoring the pressure inside the cell and inspecting the absorption spectra, was found to be negligible over a period longer than that needed to obtain a spectrum. The gas sample of F_2_C=CHF (99% pure), supplied by Peninsular Chemical Research, Inc., was used without further purification.

## 3 Computational Details

The equilibrium structure of HFO-1123 was determined according to two different composite schemes relying on the CCSD(T) method. The first one exploits the additivity relation on the energy gradient used in the geometry optimization:^53,54^
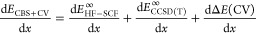
1where  and  are the energy gradients corresponding to the exp(−*Cn*) extrapolation to the complete basis set limit (CBS) at the Hartree–Fock self-consistent field level^55^ and to the *n*^–3^ CBS extrapolation for the correlation contribution,^56^ respectively. In these expressions, *n* represents the cardinal number of the Dunning’s family of correlation consistent basis sets, and specifically, the HF-SCF extrapolation was carried out using the cc-pV*n*Z with *n* = *T*, *Q*, and 5,^57,58^ while *n* = *T* and *Q* were employed for the correlation contribution. The third term, , which accounts for core–valence correlation effects, was computed as the difference between all-electron (a.e.) and frozen core (f.c.) terms computed using the core–valence cc-pCVTZ basis set.^59^ The second composite method relies on the cheap scheme (ChS), where the additivity relation is applied directly on the value of the geometrical parameter.^60,61^ Briefly, it starts from the geometry optimized at the CCSD(T)/cc-pVTZ level and, on top of it, adds contributions accounting for the extrapolation to the CBS and core–valence correlations, evaluated according to the second-order Møller–Plesset (MP2) perturbation theory^62^ in order to limit the computational cost. The best estimates of the harmonic vibrational frequencies were also obtained by applying the ChS, while for IR intensities and centrifugal distortion constants, the following expression was employed:^63^
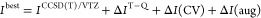
2where *I*^CCSD(T)/VTZ^ is the harmonic IR intensity (or the centrifugal distortion constant) obtained at the CCSD(T)/cc-pVTZ level; Δ*I*^T–Q^ represents the correction due to the enlargement of the basis set obtained as the difference between the MP2 values obtained with the cc-pVQZ and cc-pVTZ basis; Δ*I*(aug) takes into account the effects of diffuse functions, which are particularly relevant for intensity calculations (it is calculated as the difference between MP2 values obtained with the aug-cc-pVTZ and cc-pVTZ basis sets); Δ*I*(CV) has its usual meaning. All the required harmonic force fields were obtained through analytic evaluation of the Hessian matrix.

On the basis of the recent literature,^64,65^ full anharmonic calculations were carried out at the DFT level by adopting the double-hybrid B2PLYP^66^ and rev-DSDPBEP86^67^ functionals coupled to the aug-cc-pVTZ and jun-cc-pVTZ basis sets^68^ as well as the PW6B95 functional^69^ in conjunction with the jul-cc-pVDZ basis set.^68^ All the functionals were corrected for dispersion correlation effects by means of the Grimme’s DFT-D3 method^70^ with the use of the Beck-Johnson damping.^71^ Cubic, and semidiagonal quartic force constants, and up to the third-derivatives of the dipole moment were obtained by numerical differentiation of displaced analytic Hessians and electric dipole moment first derivatives along the normal coordinates, respectively. Vibrational corrections to rotational constants, vibrational wavenumbers, and IR intensities beyond the double-harmonic approximation were obtained by applying vibrational perturbation theory to second-order (VPT2)^72−74^ on the anharmonic force field. To improve the accuracy of the computed vibrational frequencies, a hybrid force field^36,65^ was devised by mixing the harmonic vibrational wavenumbers evaluated in the frame of the ChS with the cubic- and quartic-force constants computed by means of double-hybrid functionals. In order to tackle the problem of resonances plaguing the VPT2 approach, generalized second-order vibrational perturbation theory (GVPT2) was adopted in which (near-) singular terms are removed from the perturbative summations (leading to the so-called deperturbed approach) and the energy levels coupled by the resonances are treated in a second step by a proper variational calculation of reduced dimensionality.^75,76^ All DFT and MP2 calculations were performed with the Gaussian 16 quantum chemical package,^77^ while for CCSD(T) computations, the CFOUR program^78^ was employed. Spectroscopic parameters were evaluated according to the general VPT2 engine, implemented within the Gaussian software, and IR intensities evaluated up to three quanta of vibrational excitation.^79^

## 4 Results and Discussion

The F_2_C=CHF molecule is a planar near-prolate asymmetric rotor belonging to the *C*_s_ symmetry point group. It has 12 normal modes of vibration classified in terms of symmetry species, as 9*A*′ ⊕ 3*A*″, with *A*′ vibrations producing A/B hybrid bands with different contributions of the components and *A*″ vibrations giving rise C-type band envelopes.

### 4.1 Equilibrium Geometry and Rotational Spectroscopic Parameters

**Figure 1:**
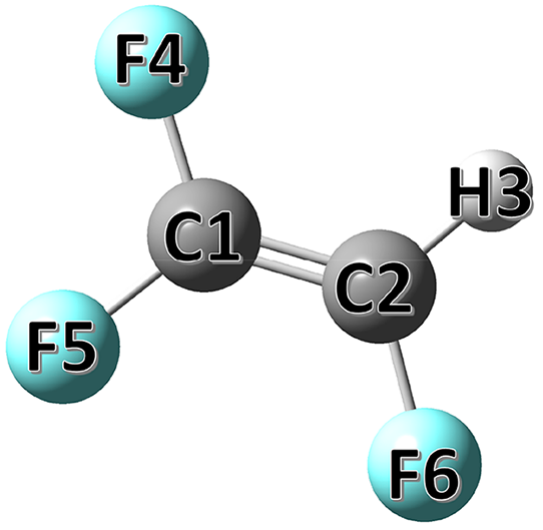
Molecular structure of HFO-1123 with atom labeling.

The HFO-1123 equilibrium structure is completely defined by nine structural parameters that are reported in Table 1 at different levels of theory with the labeling of the atoms as illustrated in Figure 1. More precisely, the table shows the results obtained from both the CCSD(T)/CBS+CV and the ChS and, for the latter, the f.c.-CCSD(T)/cc-pVTZ results are reported together with the CBS and CV corrections. As it can be seen from the results, the CCSD(T)/CBS+CV and the ChS yield the same predictions of the structural parameters with differences within 0.5 mÅ for bond lengths and 0.04° for bond angles. The expected accuracy for geometrical parameters obtained from the CCSD(T)/CBS+CV methods is 0.001–0.002 Å for distances and 0.05–0.1° for angles.^36,53,54^ An inspection of the table reveals that the CCSD(T)/cc-pVTZ and the double-hybrid functionals yield predictions of the equilibrium geometry of similar quality with differences, measured with respect to the CCSD(T)/CBS+CV structure, up to 0.007 Å and about 0.3° for distances and angles, respectively, while at the PW6B95, deviations increase slightly. The ΔCBS and ΔCV contributions are significant for the improvement of the CCSD(T)/cc-pVTZ accuracy. This is particularly true for bond lengths, systematically overestimated at the CCSD(T)/cc-pVTZ level (between 0.002 and 0.007 Å), for which both contributions are negative and range from −1 to −4 mÅ. Less systematic are the corrections for the bond angles for which the ΔCBS term ranges between −0.3° (∠ C_1_C_2_F_6_) and 0.4° (C_1_C_2_H_3_), while the ΔCV contribution is almost negligible. A comment is deserved concerning the comparison to the experimental structure derived by Mom et al.^40^ When this geometry is compared against theoretical predictions, huge differences as large as 0.025 Å (C_2_—H_3_) and −1.1° (∠ C_2_C_1_F_5_) are noticed. In part, this is due to the fact that ref (40) reports an effective structure obtained from the fitting of the ground state rotational constants and, therefore, contaminated by the vibrational motion. Second and most importantly, because of the insufficient number of isotopic substitutions, limited to the main isotopologue and the deuterated species, the effective geometry was obtained by resorting to a number of approximations, like the equivalency of the C_1_—F_4_ and C_1_—F_5_ bond lengths, which is clearly not the case differing by about 6 mÅ. As mentioned above, the rev-DSDPBEP86 functional overestimates all the bond lengths. Hence, to improve its predictions the recently proposed Nano-LEGO^80^ tool has been applied, and the results are also reported in Table 1. The Nano-LEGO augmented geometrical parameters are in an impressive agreement with those obtained by the most refined (and computationally demanding) composite schemes with deviations well within 0.001 Å from the CCSD(T)/CBS+CV bond lengths and a maximum difference of 0.2° for C_1_C_2_F_6_, which however, has not been corrected due to the lack of Nano-LEGO parametrization for this angle.

**Table 1:** Equilibrium Structure of HFO-1123^a^

	CCSD(T)/CBS+CV	CCSD(T)^b^	ΔCBS^c^	ΔCV^d^	ChS^e^	B2PLYP^f^	rDSD^g^	rDSD+NL^h^	PW6^i^	exp.^l^
C_1_=C_2_	1.3223	1.3221	–0.0043	–0.0024	1.3221	1.3224	1.3253	1.3229	1.3221	1.341(12)
C_2_—H_3_	1.0750	1.0745	–0.0012	–0.0011	1.0745	1.0750	1.0774	1.0749	1.0815	1.100(10)
C_1_—F_4_	1.3138	1.3137	–0.0030	–0.0017	1.3137	1.3203	1.3184	1.3143	1.3209	1.316(11)
C_1_—F_5_	1.3083	1.3082	–0.0036	–0.0017	1.3082	1.3147	1.3129	1.3089	1.3153	1.316(11)
C_2_—F_6_	1.3350	1.3350	–0.0024	–0.0018	1.3350	1.3417	1.3397	1.3356	1.3415	1.342(24)
∠ C_1_C_2_H_3_	123.11	123.13	0.38	–0.04	123.13	123.21	123.09	123.09	123.43	124.0(17)
∠ C_2_C_1_F_4_	122.87	122.85	0.11	0.00	122.85	122.96	122.91	122.91	123.06	123.1(15)
∠ C_2_C_1_F_5_	125.13	125.14	–0.03	–0.02	125.14	125.21	125.18	125.18	125.20	124.0(6)
∠ C_1_C_2_F_6_	120.22	120.26	–0.32	0.03	120.26	120.47	120.44	120.44	120.35	120.0(7)

a Bond lengths and angles in Å and deg, respectively.

b f.c.-CCSD(T)/cc-pVTZ level of theory.

c Correction due to the extrapolation to the CBS using MP2 theory with the cc-pVQZ and cc-pVTZ basis sets.

d Correction due to core-correlation effects.

e Best estimate according to the ChS, i.e., CCSD(T)+ΔCBS+ΔCV.

f B2PLYP-D3/aug-cc-pVTZ.

g rev-DSDPBEP86-D3/jun-cc-pVTZ.

h rev-DSDPBEP86-D3/jun-cc-pVTZ augmented through Nano-LEGO. CCF angles are not corrected due to the lack of parametrization.

i PW6B95-D3/jul-cc-pVDZ.

l From ref (40); figures in parentheses are uncertainties referred to the last significant digits.

The spectroscopic parameters relevant for rotational spectroscopy, specifically equilibrium and ground state rotational constants as well as quartic centrifugal distortion terms, are summarized in Table 2, where theoretical predictions are compared against the experimental data in the *I*^*r*^ representation of the Watson’s *A* reduced Hamiltonian.^81^ A well consolidated procedure was followed,^30,38,80^ and the best estimates of ground state rotational constants have been obtained by augmenting those of the equilibrium configuration computed according to the CCSD(T)/CBS+CV composite scheme through rev-DSDPBEP86-D3/jun-cc-pVTZ vibrational corrections. The resulting parameters are in very good agreement with the corresponding experimental counterparts with discrepancies of 0.06%, thus confirming the accuracy of the CCSD(T)/CBS+CV equilibrium geometry as well as the reliability of the rev-DSDPBEP86 vibrational contributions. Analogous results (not reported) have been obtained using both the ChS and the Nano-LEGO augmented rev-DSDPBEP86 equilibrium rotational constants. The accuracy of the ground state rotational constants evaluated at the CCSD(T)/cc-pVTZ, B2LYP-D3/aug-cc-pVTZ, and rev-DSDPBEP86-D3/jun-cc-pVTZ levels of theory are similar with rev-DSDPBEP86 providing slightly better results and mirror that of the geometry: the three methods underestimate the experimental values and show deviations around −0.9%, −0.7%, and −0.6%, respectively. Upon inspection of Table 2, it can be observed that CCSD(T)/cc-pVTZ and the double-hybrid computations also systematically underestimate quartic centrifugal parameters with absolute deviations in the ranges of 1.1–6.7%, 0.6–3.2%, and 0.7% – 3.6% respectively, an accuracy coherent with previous benchmark studies.^64,65,82^ The ChS results reproduce quartic centrifugal distortion parameters very well, the absolute percentage deviations being in the range of 0.3–2.6%, thus sensibly improving the CCSD(T)/cc-pVTZ outcomes in all cases but the Δ_JK_ term.

**Table 2:** Rotational and Quartic Centrifugal Distortion Constants of HFO-1123 (MHz)

	best estimate^a^	CCSD(T)^b^	B2PLYP^c^	rev-DSD^d^	exp.^e^
*A*_e_	10 723.27	10 626.84	10 650.43	10 660.92	
*B*_e_	3887.73	3850.17	3855.48	3857.31	
*C*_e_	2853.27	2826.22	2830.73	2832.47	
*A*_0_	10 671.89	10 575.09	10 599.04	10 609.33	10 665.481287(51)
*B*_0_	3874.60	3836.81	3842.35	3844.15	3872.406579(24)
*C*_0_	2839.57	2812.40	2817.03	2818.74	2837.960953(29)
Δ_J_ × 10^3^	0.7285	0.7075	0.7111	0.7099	0.731145(12)
Δ_JK_ × 10^3^	7.8681	7.5942	7.6282	7.6187	7.671250(52)
Δ_K_ × 10^3^	4.8572	4.6008	4.8030	4.7438	4.92912(16)
δ_J_ × 10^3^	0.1811	0.1769	0.1775	0.1772	0.1831457(50)
δ_K_ × 10^3^	4.8543	4.7171	4.7329	4.7288	4.83607(14)

a CCSD(T)/CBS+CV equilibrium rotational constants, rev-DSDPBEP86-D3/jun-cc-pVTZ vibrational corrections, and ChS quartic centrifugal distortion constants.

b CCSD(T)/cc-pVTZ.

c B2PLYP-D3/aug-cc-pVTZ.

d rev-DSDPBEP86-D3/jun-cc-pVTZ

e From ref (42); values refer to the A-reduction Watson’s Hamiltonian in the *I*^r^ representation. The figures in parentheses are uncertainties referred to the last significant digits.

The sextic centrifugal distortion constants computed at different levels of theory are reported in Table 3 together with those determined experimentally in ref (41) and more recently by Tamassia et al.^42^ within the *I*^r^ representation of the Watson’s *A* reduced Hamiltonian.^81^ The inspection of this table reveals huge discrepancies between the two sets of experimental values, even though sextic centrifugal distortion parameters determined in ref (41) suffer from large uncertainties and, in practice, cannot be considered well determined. On the other side, according to the recent literature on the subject,^83,84^ the computed sextic centrifugal distortion constants are expected to have an average accuracy of around 10%, which is indeed fully matched by the more recent experimental values^42^ with which the average agreement is around 5% for both the functionals, and the maximum discrepancies, reported for the ϕ_J_ term, amount to 10% and 13% at the B2PLYP and rev-DSDPBEP86-D3 levels, respectively. Therefore, the present theoretical outcomes confirm the reliability of the ground state rotational centrifugal distortion terms measured by Tamassia et al.^42^

**Table 3:** Theoretical Sextic Centrifugal Distortion Constants (A-Reduction Watson’s Hamiltonian in the *I*^r^ Representation) of HFO-1123 (Hz) and Comparison to Experimental Values

	B2PLYP^a^	rev-DSD^b^	exp.^c^	exp.^d^
Φ_J_ × 10^3^	0.214	0.207	0.038(20)	0.2010(29)
Φ_JK_	0.02064	0.02011	0.0027(105)	0.019977(56)
Φ_KJ_	–0.05331	–0.05313	0.0025(288)	–0.05529(18)
Φ_K_	0.07050	0.07076	0.033(21)	0.07534(20)
ϕ_J_ × 10^3^	0.06858	0.06659	–0.02(18)	0.0765(15)
ϕ_JK_ × 10^3^	10.04	9.77	14.4(63)	9.352(55)
ϕ_K_	0.11355	0.11277	–0.041(78)	0.11635(37)

a B2PLYP-D3/aug-cc-pVTZ.

b rev-DSDPBEP86-D3/jun-cc-pVTZ.

c From ref (41); figures in parentheses are uncertainties referred to the last significant digits.

d From ref (42); figures in parentheses are uncertainties referred to the last significant digits.

Before moving to the characterization of the vibrational properties of HFO-1123, the attention is briefly moved to the ro-vibrational spectroscopic parameters. In particular, Table 4 shows the best estimates of the rotational constants for the excited fundamental vibrational levels obtained by correcting the best estimated equilibrium rotational constants through vibrational contributions evaluated at the rev-DSDPBEP86-D3/jun-cc-pVTZ. It should be noted that, according to the VPT2 framework, the vibrational dependence of the rotational constants is ruled by the α_k_^β^ vibration–rotation interaction constants that, for the sake of completeness, are reported in Table 5. The rotational constants of *v*_3_ = 1, *v*_4_ = 1, *v*_5_ = 1, *v*_6_ = 1, *v*_8_ = 1, *v*_9_ = 1, and *v*_12_ = 1 vibrational levels evaluated theoretically can be compared with those determined experimentally,^42,45−47^ which are listed as well in Table 4 within the parentheses. For these, a very good agreement is noted between the predicted and measured values with errors in all cases well within 0.1% with the only exception of the *A* rotational constant of the *v*_9_ = 1 and *v*_5_ = 1 levels. While the former deviates by only 0.12% from the experimental counterpart, the latter show a larger discrepancy, around 0.3%. It is worthwhile to note, however, that the experimental value may be affected by spurious Coriolis and anharmonic interactions. It can be concluded that the rotational constants reported in Table 4 almost retain the same accuracy as that reached for the ground state rotational constants, thus highlighting the reliability of the computed α_k_^β^ constants, and hence, they can be employed as a guide for future high-resolution investigations on this molecule. For this purpose, the most relevant Coriolis coupling constants computed at the rev-DSDPBEP86-D3/jun-cc-pVTZ are also given in Table 6.

**Table 4:** Rotational Constants (MHz) of the Excited Fundamental Vibrational Levels of HFO-1123^a^

vibrational level	*A*^v^	*B*^v^	*C*^v^
*v*_1_ = 1	10 654.68	3871.27	2836.66
*v*_2_ = 1	10 646.88	3861.08	2831.09
*v*_3_ = 1	10 647.42 (10 638.50)	3874.15 (3871.10)	2836.39 (2834.17)
*v*_4_ = 1	10 655.46 (10 652.71)^b^	3864.76 (3863.89)^b^	2830.22 (2829.71)^b^
*v*_5_ = 1	10 682.59 (10 651.20)^c^	3869.29 (3867.49)^c^	2837.08 (2833.86)^c^
*v*_6_ = 1	10 647.03 (10 641.54)^d^	3869.98 (3867.84)^d^	2833.37 (2831.90)^d^
*v*_7_ = 1	10 681.78	3878.58	2840.14
*v*_8_ = 1	10 681.66 (10 673.41)^e^	3876.78 (3874.51)^e^	2837.35 (2835.74)^e^
*v*_9_ = 1	10 637.11 (10 624.33)^e^	3873.82 (3870.68)^e^	2837.47 (2835.67)^e^
*v*_10_ = 1	10 671.26	3874.57	2842.42
*v*_11_ = 1	10 656.06	3872.38	2841.43
*v*_12_ = 1	10 699.53 (10 698.10)^e^	3880.83 (3879.12)^e^	2843.05 (2841.51)^e^

a Predicted values obtained by correcting CCSD(T)/CBS+CV equilibrium rotational constants through vibrational contributions at rev-DSDPBEP86-D3/jun-cc-pVTZ. Reported in parentheses are experimental values.

b From ref (45).

c From ref (46).

d From ref (47).

e From ref (42).

**Table 5:** α_k_^β^ Vibrational Interaction Constants (MHz) of HFO-1123 Evaluated at the rev-DSDPBEP86-D3/jun-cc-pVTZ Level of Theory

normal mode	*a*	*b*	*c*
1	17.326	3.217	2.859
2	25.120	13.421	8.431
3	24.577	0.352	3.129
4	16.551	9.740	9.309
5	–10.596	5.198	2.431
6	24.963	4.528	6.157
7	–9.784	–4.101	–0.635
8	–9.648	–2.295	2.164
9	34.886	0.689	2.041
10	0.734	–0.070	–2.911
11	15.952	2.129	–1.924
12	–27.518	–6.346	–3.534

**Table 6:** Relevant Coriolis Coupling Constants of HFO-1123 Evaluated at the rev-DSDPBEP86-D3/jun-cc-pVTZ Level of Theory

A-type Coriolis			B-type Coriolis			C-type Coriolis		
mode k	mode l	|ζ_kl_^a^|	mode k	mode l	|ζ_kl_^b^|	mode k	mode l	|ζ_kl_^c^|
1	10	0.967	1	10	0.106	1	2	0.286
1	11	0.147	2	10	0.457	1	3	0.645
1	12	0.106	2	11	0.739	1	4	0.610
2	11	0.413	2	12	0.162	1	5	0.278
3	10	0.139	3	10	0.632	1	6	0.178
3	11	0.713	3	12	0.137	1	9	0.117
3	12	0.135	4	10	0.564	2	3	0.549
4	11	0.347	4	11	0.572	2	4	0.401
4	12	0.152	4	12	0.401	2	5	0.460
5	11	0.361	5	10	0.168	2	6	0.246
5	12	0.466	5	11	0.121	2	7	0.366
6	10	0.165	5	12	0.696	2	8	0.191
6	12	0.302	6	10	0.124	2	9	0.107
7	11	0.118	6	12	0.339	3	4	0.325
7	12	0.459	7	11	0.248	3	7	0.310
8	11	0.165	8	11	0.106	3	8	0.230
8	12	0.377	8	12	0.122	3	9	0.110
9	12	0.533	9	10	0.127	4	6	0.188
			9	11	0.182	4	7	0.247
			9	12	0.423	4	8	0.453
						4	9	0.236
						5	6	0.457
						5	7	0.332
						5	9	0.586
						6	7	0.161
						6	8	0.147
						6	9	0.394
						7	8	0.518
						8	9	0.556

### 4.2 Harmonic Vibrational Frequencies and IR Intensities

Harmonic wavenumbers and IR intensities calculated within the double-harmonic approximation at different levels of theory are summarized in Tables 7 and 8, respectively. In addition to the best-estimated values from the ChS method, these tables also list its different contributions as well as predictions from the B2PLYP-D3, rev-DSDPBEP86-D3, and PW6B95-D3 density functionals. Concerning wavenumbers, corrections due to the CBS extrapolation are always negative, and they range between −1 and −7 cm^–1^ with the exception of ω_3_, ω_4_, and ω_5_ for which the contributions are −20, −16, and −12 cm^–1^, respectively. Diffuse functions also provide a negative contribution between −2.7 cm^–1^ (ω_9_) and −19.7 cm^–1^ (ω_3_) and usually are of a larger magnitude than the ΔCBS term. Conversely, the core–valence correlation yields positive corrections, generally of smaller magnitude, being 6 cm^–1^ at most in the case of the ω_2_ normal mode. Summed up, the different contributions cause a blue-shift of the CCSD(T)/cc-pVTZ harmonic wavenumbers for all the vibrational normal modes, which reaches −36.6 and −30.1 cm^–1^ for the ω_3_ and ω_4_ vibrations, respectively. Concerning the intensities, all three corrections can be either positive or negative and, in absolute terms, they are generally small. In particular, core–valence contributions are always lower than 0.7 km mol^–1^, and in general, they are almost negligible. Likewise, the Δ*I*(aug) term is always lower than 0.8 km mol^–1^ with exceptions being given by ω_4_ and ω_5_ for which it amounts to 8.1 and 5.1 km mol^–1^, respectively, and ω_1_ for which it is 1.6 km mol^–1^. The latter is small in absolute value but, in relative terms, it represents 14.1% of the overall ChS harmonic IR intensity for this normal mode. The largest CBS correction, for the ω_4_ vibration, is around 9 km mol^–1^, which corresponds to only 5% of the overall intensity. Conversely, the CBS correction for ω_1_ amounts to 1.9 km mol^–1^, which accounts for almost 17% of the ChS IR intensity.

**Table 7:** Approximate Description and Harmonic Wavenumbers (cm^–1^) Obtained at Different Levels of Theory for the HFO-1123 Vibrational Normal Modes

sym.	mode	approximate description	ω^CCSD(T)^^a^	Δω[CBS(T,Q)]^b^	Δω(CV)^c^	Δω(aug)^d^	ω^ChS^^e^	ω^B2^^f^	ω^rDSD^^g^	ω^PW6^^h^
*A*′	ω_1_	CH stretch	3272.0	–0.3	5.1	–4.5	3272.3	3279.9	3277.4	3296.9
	ω_2_	CC stretch	1836.3	–6.7	6.0	–11.3	1824.3	1827.9	1837.1	1871.2
	ω_3_	CH bend + CF_2_ asym. stretch	1402.4	–20.4	3.5	–19.7	1365.8	1373.1	1384.2	1365.2
	ω_4_	CF_2_ asym. stretch + CH bend	1301.5	–16.4	3.3	–16.9	1271.4	1274.1	1286.2	1270.3
	ω_5_	CF stretch (CHF) + CH bend	1196.1	–12.3	3.2	–13.9	1173.1	1175.4	1185.1	1180.4
	ω_6_	CF_2_ sym. stretch + CH bend	944.1	–3.1	2.8	–6.3	937.5	941.9	945.8	944.9
	ω_7_	CHF rock + CF_2_ scissor	628.8	–2.7	2.0	–5.0	623.2	626.6	628.9	622.4
	ω_8_	CF_2_ scissor	488.9	–1.8	1.6	–4.8	484.0	486.5	488.1	480.9
	ω_9_	CF_2_ rock	232.9	–1.5	0.9	–2.7	229.6	233.1	233.1	225.2
*A*″	ω_10_	CHF wag	771.2	–7.0	4.1	–16.3	752.0	778.7	783.1	783.0
	ω_11_	CF_2_ wag	564.6	–2.6	3.3	–11.6	553.7	579.4	583.4	586.7
	ω_12_	C=C torsion	310.3	–1.8	1.0	–4.4	305.0	310.1	313.0	310.8

a f.c.-CCSD(T)/cc-pVTZ.

b CBS contribution based on MP2 computations with cc-pVTZ and cc-pVTZ basis sets.

c Correction due to core-correlation effects.

d Contribution from diffuse functions.

e Best estimate according to ChS, i.e., CCSD(T)+Δ[CBS(T,Q)]+Δ(CV)+Δ(aug).

f B2PLYP-D3/aug-cc-pVTZ.

g rev-DSDPBEP86-D3/jun-cc-pVTZ.

h PW6B95-D3/jul-cc-pVDZ.

**Table 8:** Harmonic Intensities (km mol^–1^) Obtained at Different Levels of Theory for the HFO-1123 Vibrational Normal Modes

sym.	mode	*I*^CCSD(T)^^a^	Δ*I*[CBS(T,Q)]^b^	Δ*I*(CV)^c^	Δ*I*(aug)^d^	*I*^ChS^^e^	*I*^B2^^f^	*I*^rDSD^^g^	*I*^PW6^^h^
*A*′	ω_1_	7.76	1.90	0.13	1.60	11.38	10.49	9.91	11.44
	ω_2_	66.47	1.17	0.14	0.79	68.58	77.97	76.03	79.56
	ω_3_	121.93	–2.75	0.63	–0.10	119.71	105.19	117.09	136.21
	ω_4_	179.22	9.48	–0.39	8.10	196.41	205.94	197.56	200.12
	ω_5_	131.68	3.63	–0.05	5.11	140.38	161.13	150.40	145.04
	ω_6_	55.98	0.82	0.12	0.09	57.01	58.57	57.84	60.37
	ω_7_	3.50	0.18	–0.02	0.25	3.90	3.36	3.57	3.51
	ω_8_	2.12	–0.05	–0.01	0.05	2.11	1.84	1.91	2.09
	ω_9_	4.11	–0.15	–0.01	–0.11	3.85	3.94	3.97	3.97
*A*″	ω_10_	30.64	–0.44	0.21	0.05	30.46	34.11	33.18	33.43
	ω_11_	0.44	–0.33	–0.06	–0.19	0.00	0.00	0.01	0.00
	ω_12_	4.24	0.13	0.03	–0.09	4.31	4.02	3.98	4.42

a f.c.-CCSD(T)/cc-pVTZ.

b CBS contribution based on MP2 computations with cc-pVTZ and cc-pVTZ basis sets.

c Correction due to core-correlation effects.

d Contribution from diffuse functions.

e Best estimate according to ChS, i.e., CCSD(T)+Δ[CBS(T,Q)]+Δ(CV)+Δ(aug).

f B2PLYP-D3/aug-cc-pVTZ.

g rev-DSDPBEP86-D3/jun-cc-pVTZ.

h PW6B95-D3/jul-cc-pVDZ.

When the ChS best estimates are taken as the reference, it is seen that the double-hybrid functionals yield, on the whole, similar predictions with a mean absolute deviation (MAD) from ChS harmonic frequencies of 6.7 and 4.0 cm^–1^ at the B2PLYP-D3/aug-cc-pVTZ and rev-DSDPBEP86-D3/jun-cc-pVTZ levels of theory, respectively. For the former, the maximum deviations, around 14 cm^–1^ but in opposite directions, are observed for ω_4_ and ω_11_, which is also responsible for the largest deviation (18 cm^–1^) observed among the rev-DSDPBEP86-D3 harmonic frequencies. This functional shows a better agreement to the ChS harmonic intensities than B2PLYP-D3. Indeed, the former presents a MAD of c.a. 2 km mol^–1^ and maximum discrepancies in the range of −2.6–10 km mol^–1^, while the latter reproduces the best estimates with a MAD of 5 km mol^–1^ and deviations between −14.5 and 20.8 km mol^–1^ for the intensities of normal modes 3 and 5, respectively. As expected, the hybrid PW6B95-D3 functional shows slightly larger deviations for harmonic wavenumbers with a MAD of around 13 cm^–1^ and maximum deviation of about 35 cm^–1^ for ω_2_. On the other hand, it well reproduces ChS harmonic intensities with the MAD being 3.6 km mol^–1^.

### 4.3 Anharmonic Force Field and Characterization of the Gas-Phase Spectra

Fundamental vibrational frequencies calculated at different levels of theory are presented in Table 9, where they are also compared to the wavenumbers measured experimentally, while theoretical IR intensities beyond the double-harmonic approximation are collected in Table 10. Following the outcomes obtained for harmonic frequencies and intensities, in addition to full anharmonic computations at the rev-DSDPBEP86-D3/jun-cc-pVTZ and PW6B95-D3/jul-cc-pVDZ, a hybrid force field, termed ChS:rDSD, has been worked out. This has harmonic frequencies estimated by the ChS method mixed with cubic and semidiagonal quartic force constants evaluated at the rev-DSDPBEP86-D3 level, while hybrid anharmonic intensities have been obtained from ChS and rev-DSDPBEP86-D3 contributions, according to eq 2. According to the available literature, the adopted hybrid force field is expected to predict fundamental transition frequencies with an average accuracy within 5 cm^–1^, a maximum error of 10 cm^–1^, and anharmonic IR intensities with an error of a few km mol^–1^; hence, ChS:rDSD simulations have been used to guide and support the analysis of the observed spectra.^30,36,38,64,65^

**Table 9:** Experimental and Theoretical Fundamental Wavenumbers (cm^–1^) of the HFO-1123 Normal Modes of Vibration

symmetry	mode	exp.	ChS:rDSD^a^	rDSD^b^	PW6^c^
*A*′	ν_1_	3163.65	3174 (3148)^d^	3176	3187
	ν_2_	1787.42	1794	1796	1825
	ν_3_	1360.8	1357	1357	1336
	ν_4_	1264.78	1261	1259	1244
	ν_5_	1172.41	1171 (1165)^d^	1172	1167
	ν_6_	929.5	929	931	931
	ν_7_	623.83	624	624	620
	ν_8_	484.93	485	484	477
	ν_9_	232.9	232	233	228
*A*″	ν_10_	750.59	749	764	770
	ν_11_	553.76	554	573	576
	ν_12_	305.1	304	307	306
ME^e^			–0.4	–3.9	–3.1
MAE^f^			2.6	5.5	14.6
max. neg.^g^			–10.4	–19.2	–37.9
max. pos.^h^			3.8	5.3	20.8

a Hybrid anharmonic wavenumbers obtained by mixing ChS harmonic properties with cubic and semidiagonal quartic force constants at the rev-DSDPBEP86-D3/jun-cc-pVTZ level.

b rev-DSDPBEP86-D3/jun-cc-pVTZ.

c PW6B95-D3/jul-cc-pVDZ.

d Deperturbed value within parentheses.

e Mean error.

f Mean absolute error.

g Maximum negative error.

h Maximum positive error.

**Table 10:** Theoretical Anharmonic IR Intensities (km mol^–1^) of the HFO-1123 Fundamental Transitions

symmetry	mode	ChS:rDSD^a^	rDSD^b^	PW6^c^
*A*′	ν_1_	6.63	5.16	6.33
	ν_2_	62.42	69.88	60.49
	ν_3_	113.34	110.72	128.16
	ν_4_	175.67	176.82	171.39
	ν_5_	51.36	61.38	54.09
	ν_6_	54.94	55.77	58.00
	ν_7_	3.19	2.87	2.39
	ν_8_	2.05	1.86	2.02
	ν_9_	3.86	3.98	3.98
*A*″	ν_10_	29.27	31.98	31.46
	ν_11_	0.00	0.01	0.01
	ν_12_	4.37	4.05	4.32

a Hybrid intensities obtained from ChS harmonic intensities and rev-DSDPBEP86-D3/jun-cc-pVTZ anharmonic contributions.

b rev-DSDPBEP86-D3/jun-cc-pVTZ.

c PW6B95-D3/jul-cc-pVDZ.

**Figure 2:**
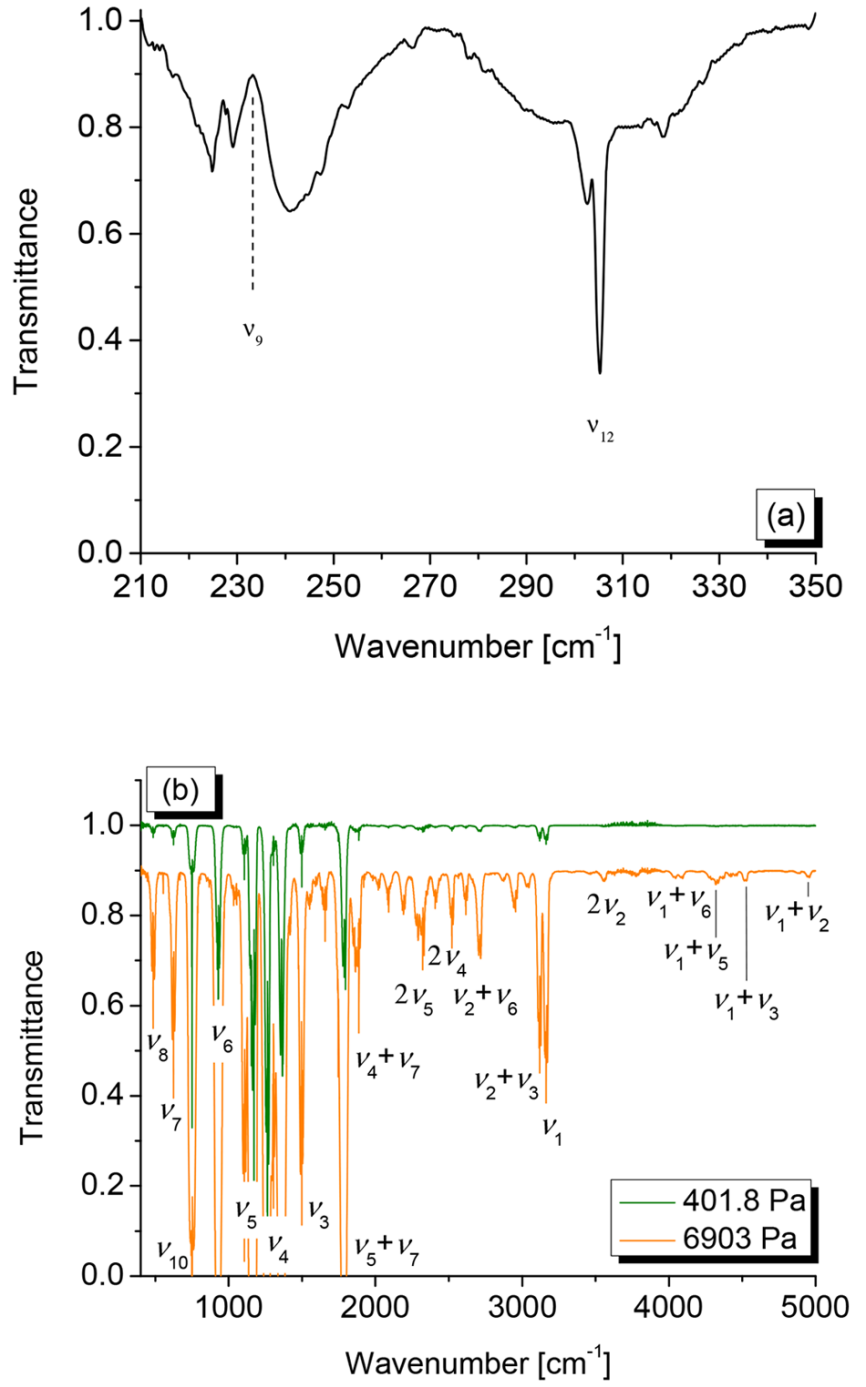
Gas-phase spectrum of HFO-1123. (a) Spectral region between 210 and 350 cm^–1^ (resolution = 0.5 cm^–1^, optical path = 15 cm, temperature = 293 K, sample pressure = 48.2 hPa). (b) Spectral region between 400 and 5000 cm^–1^ (resolution = 0.5 cm^–1^, optical path = 13.4 cm, temperature = 298 K): the upper trace (green) corresponds to a sample pressure of 401.8 Pa, and the lower trace (orange, displaced downward for clarity), corresponds to a sample pressure of 69.03 hPa.

**Figure 3:**
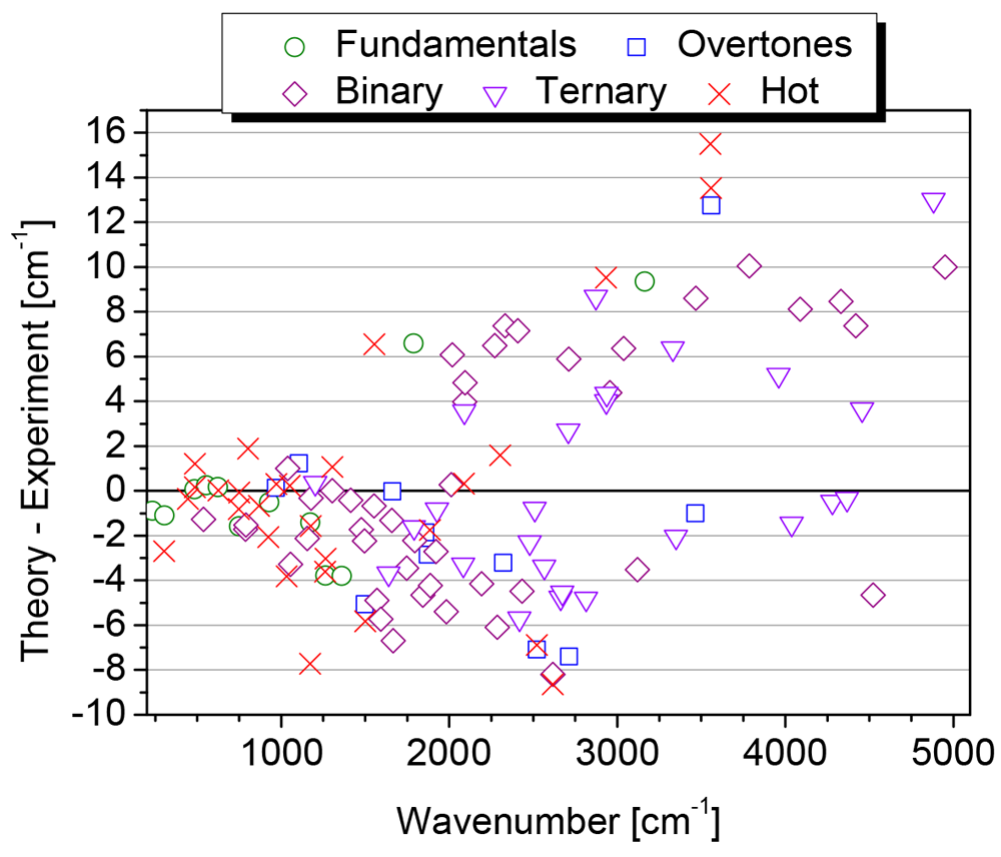
Differences between computed and observed transition frequencies as a function of the wavenumbers and type of transition for the assigned bands of HFO-1123.

Survey gas-phase IR spectra of HFO-1123 in the ranges of 200–400 cm^–1^ and 400–5000 cm^–1^ are reported in Figure 2 in which some relevant absorptions are also labeled. The former region is characterized by the absorptions of the lowest lying fundamentals: the ν_9_ vibration, corresponding to the CF_2_ rocking is located at 232.9 cm^–1^, and the ν_12_ normal mode, due to the C=C torsion, measured at 302.7 cm^–1^ in agreement with previous measurements.^43^ Both of them perfectly match the theoretical predictions of the ChS:rDSD hybrid force field that places the ν_9_ and ν_12_ transitions at 232 and 304 cm^–1^, respectively. In addition to the fundamentals, the 2ν_12_–ν_12_ hot band has been observed, as detailed in Table 11 that shows the full list of assigned vibrational transitions and where the frequencies measured experimentally are compared with those from the ChS:rDSD predictions. The comparison, in terms of differences between the theoretical and experimental values as a function of increasing wavenumbers, is illustrated in Figure 3 in which the data are reported grouped according to the transition type (i.e., fundamentals, overtones, two- and three-quanta combinations, and hot bands). As it can be seen, the almost completeness of the transitions assigned experimentally are reproduced within 10 cm^–1^, and only four bands fall outside this range. While deviations appear randomly scattered around zero, the only dependence that can be inferred to some extent is that most experimental values are underestimated below c.a. 2000 cm^–1^, a trend that reverses moving toward higher frequencies. At room temperature, the populations of the *v*_9_ = 1 and *v*_12_ = 1 levels are about 32% and 23% of the vibrational ground state; hence, a number of hot bands has to be expected in addition to cold transitions.

**Table 11:** Vibrational Assignment of HFO-1123 and Comparison to Theoretical Wavenumbers (cm^–1^)

assignment	exp.	ChS:rDSD	assignment	exp.	ChS:rDSD	assignment	exp.	ChS:rDSD
ν_9_	232.9	232	ν_2_ – ν_12_	1492.74	1491	ν_3_ + ν_6_ + ν_9_	2509.80	2509
2ν_12_ – ν_12_	302.69	300	ν_4_ + ν_9_	1495.24	1493	2ν_4_ + ν_9_ – ν_9_	2520.89	2514
ν_12_	305.1	304	2ν_10_	1498.07	1493	2ν_4_	2522.09	2515
ν_10_ – ν_12_	445.36	445	ν_5_ + ν_11_ – ν_9_	1498.83	1493	ν_4_ + ν_10_ + ν_11_	2565.40	2562
ν_8_	484.93	485	ν_6_ + ν_7_	1552.68	1552	ν_3_ + ν_4_ + ν_9_ – ν_9_	2615.64	2607
ν_9_ + ν_9_ – ν_9_	485.84	486	ν_2_ – ν_9_	1555.45	1562	ν_3_ + ν_4_	2617.20	2609
ν_8_ + 2ν_9_ – 2ν_9_	486.80	488	ν_4_ + ν_12_	1568.89	1564	ν_3_ + ν_10_ + ν_11_	2662.77	2658
ν_9_ + ν_12_	537.28	536	ν_3_ + ν_9_	1592.73	1587	ν_4_ + ν_6_ + ν_8_	2672.54	2668
ν_11_	553.76	554	ν_5_ + 2ν_9_	1639.7	1636	ν_5_ + ν_6_ + ν_7_	2709.30	2712
ν_7_	623.83	624	ν_5_ + ν_8_	1657.32	1656	ν_2_ + ν_6_	2711.11	2717
ν_11_ + ν_12_ – ν_9_	625.97	626	3ν_11_	1661.02	1661	2ν_3_	2713.40	2706
ν_9_ + ν_10_ – ν_9_	747.82	747	ν_3_ + ν_12_	1665.7	1659	ν_4_ + ν_6_ + ν_7_	2813.82	2809
ν_10_ + ν_9_ – ν_9_	749.08	749	ν_4_ + ν_8_	1746.46	1743	ν_2_ + 2ν_11_	2874.33	2883
ν_10_	750.59	749	ν_2_	1787.42	1794	ν_1_ – ν_9_	2931.47	2941
ν_9_ + ν_11_	787.72	786	ν_8_ + ν_10_ + ν_11_	1789.62	1788	2ν_5_ + ν_7_	2934.00	2938
ν_8_ + ν_12_	789.52	788	ν_5_ + ν_7_	1795.22	1793	ν_2_ + ν_6_ + ν_9_	2936.66	2941
2ν_11_ – ν_12_	802.12	804	ν_3_ + ν_8_	1843.65	1839	ν_2_ + ν_5_	2954.62	2959
ν_5_ – ν_12_	867.67	867	2ν_6_	1856.86	1855	ν_2_ + ν_4_	3038.64	3045
ν_6_ + ν_9_ – ν_9_	923.06	921	3ν_7_	1871.86	1869	ν_2_ + ν_3_	3119.52	3116
ν_6_	929.51	929	ν_4_ + ν_6_ – ν_12_	1885.75	1884	ν_1_	3163.65	3173
2ν_8_	969.86	970	ν_4_ + ν_7_	1887.23	1883	ν_2_ + ν_6_ + ν_7_	3329.64	3336
2ν_8_ + ν_9_ – ν_9_	971.70	972	ν_5_ + ν_10_	1920.71	1918	ν_4_ + ν_5_ + ν_6_	3349.05	3347
ν_4_ – ν_9_	1032.82	1029	ν_7_ + ν_10_ + ν_11_	1926.83	1926	3ν_5_	3465.01	3464
ν_8_ + ν_11_	1038.00	1039	ν_3_ + ν_7_	1982.40	1977	ν_1_ + ν_12_	3468.40	3477
ν_10_ + ν_12_ + ν_9_ – ν_9_	1050.78	1051	ν_4_ + ν_10_	2010.73	2011	2ν_2_ + ν_12_ – ν_12_	3553.50	3569
ν_10_ + ν_12_	1054.28	1051	ν_2_ + ν_9_	2018.93	2025	2ν_2_ + ν_9_ – ν_9_	3557.47	3571
2ν_11_	1106.78	1108	2ν_6_ + ν_9_	2083.30	2080	2ν_2_	3560.25	3573
ν_6_ + ν_9_	1155.12	1153	2ν_5_ – ν_12_	2086.68	2087	2ν_3_ + ν_6_	3630.04	3622
ν_5_ + ν_12_ – ν_12_	1170.73	1163	ν_5_ + ν_7_ + ν_12_	2089.45	2093	ν_1_ + ν_7_	3784.95	3795
ν_5_	1172.41	1171	ν_2_ + ν_12_	2091.02	2095	ν_2_ + ν_4_ + ν_6_	3960.83	3966
ν_6_ + ν_11_ – ν_12_	1175.58	1174	ν_5_ + ν_6_	2093.18	2098	ν_2_ + ν_3_ + ν_6_	4038.46	4037
ν_7_ + ν_11_	1178.32	1178	ν_4_ + ν_6_	2192.15	2188	ν_1_ + ν_6_	4087.88	4096
2ν_8_ + ν_9_	1203.64	1204	ν_2_ + ν_8_	2270.51	2277	ν_2_ + ν_3_ + ν_5_	4281.48	4281
ν_4_ + ν_8_ – ν_8_	1261.62	1258	ν_3_ + ν_6_	2285.10	2279	ν_1_ + ν_5_	4330.54	4339
ν_4_ + ν_12_ – ν_12_	1264.03	1261	ν_3_ + ν_4_ – ν_12_	2303.42	2305	ν_2_ + ν_3_ + ν_4_	4366.35	4366
ν_4_	1264.78	1261	2ν_5_	2322.22	2319	ν_1_ + ν_4_	4419.63	4427
ν_8_ + ν_10_ + ν_12_ – ν_9_	1302.94	1304	ν_2_ + ν_11_	2331.63	2339	ν_2_ + 2ν_3_	4457.36	4461
ν_10_ + ν_11_	1304	1304	ν_2_ + ν_7_	2406.85	2414	ν_1_ + ν_3_	4523.66	4519
ν_3_	1360.80	1357	ν_4_ + ν_6_ + ν_9_	2417.70	2412	ν_2_ + 2ν_3_	4884	4897
ν_6_ + ν_8_	1413.42	1413	ν_4_ + ν_5_	2433.49	2429	ν_1_ + ν_2_	4951.00	4961
ν_5_ + ν_12_	1475.74	1474	2ν_6_ + ν_7_	2479.30	2477			

The spectral portion between 400 and 1000 cm^–1^ features a number of absorptions mainly related to the fundamental transitions ν_8_, ν_7_, ν_10_, and ν_6_ and their hot bands involving, as expected, the *v*_9_ = 1 and *v*_12_ = 1 excited levels. Among these, the most prominent ones are ν_6_, the CF_2_ symmetric stretching, and ν_10_, the CHF wagging, with a predicted intensity of 54.9 and 29.3 km mol^–1^ and located at 929.5 and 750.6 cm^–1^, respectively, in perfect agreement with quantum chemical predictions (929 and 749 cm^–1^). The CHF rocking and CF_2_ scissoring motions correspond to the ν_7_ and ν_8_ normal modes, respectively, that have similar intensities and give rise to the bands observed at 623.8 and 484.9 cm^–1^. In between these two bands, the very weak ν_11_ feature, stemming from the CF_2_ wagging motion, can be detected at 553.8 cm^–1^ only in the spectra recorded at the higher pressures, again in perfect accord with the ChS:rDSD predictions (554 cm^–1^) that significantly improves the results obtained from the rev-DSDPBEP86 and PW6B95 functionals. The fundamental transitions ν_5_, ν_4_, and ν_3_, associated, respectively, to the C–F stretching of the CHF group, the CF_2_ asymmetric stretching, and the CH in-plane bending motion cover the 1000–1700 cm^–1^ spectral portion. The ν_5_ and ν_4_ modes give rise to two A/B hybrid bands with a prevalence of A-type character located at 1172.4 and 1264.8 cm^–1^ and with a predicted intensity of 51.4 and 175.7 km mol^–1^, respectively. The *v*_5_ = 1 level is also involved in a type II Fermi interaction with the *v*_6_ = *v*_9_ = 1 level, whose corresponding combination band is observed on the low-frequency side of ν_5_ at 1155.1 cm^–1^. According to the ChS:rDSD hybrid force field, this two-level system can be described by the following interaction matrix, 
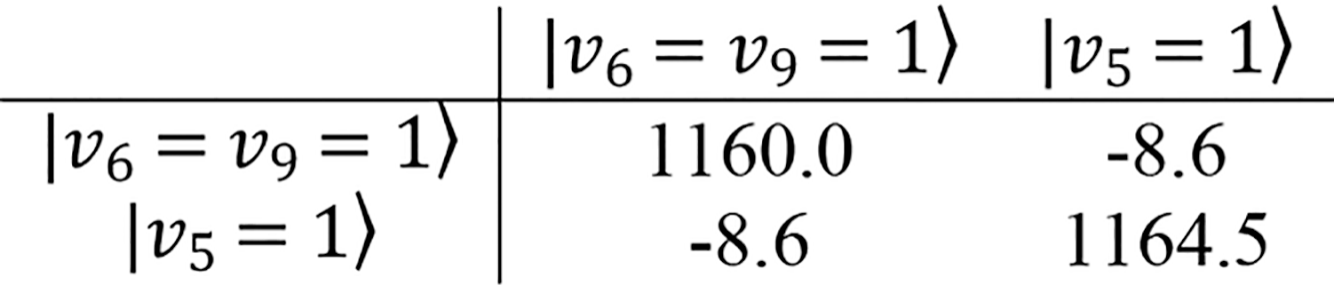
where diagonal elements are deperturbed and the off-diagonal coupling element is the cubic force constant ϕ_569_/√8. The eigenvalues of this matrix are 1153 and 1171 cm^–1^ in optimal agreement with the measured transition frequencies. The ν_3_ vibration appears at 1360.8 cm^–1^ with a B-type band envelope and an intensity (according to the ChS:rDSD computations) of 113.3 km mol^–1^. The remaining relevant absorptions in this spectral region include the first overtones of ν_11_ and ν_10_ at 1106.8 and 1498.1 cm^–1^, respectively, and the ν_10_ + ν_11_ combination measured at 1304.1 cm^–1^, all showing an A/B-type envelope with prevailing A-character. When one focuses on the portion between 1700 and 3500 cm^–1^, the strongest features come from the ν_2_ fundamental, corresponding to the stretching of the C=C double bond and measured at 1787.4 cm^–1^ and ν_1_ centered at 3163.7 cm^–1^ associated with the C–H stretching. The latter is involved in a quite strong Fermi resonance of type II with the nearby ν_2_ + ν_3_ combination appearing at 3119.5 cm^–1^ as an A/B-type band of intensity comparable to that of ν_1_. Using deperturbed frequencies obtained at the ChS:rDSD level, this resonance can be modeled by the following interaction Hamiltonian
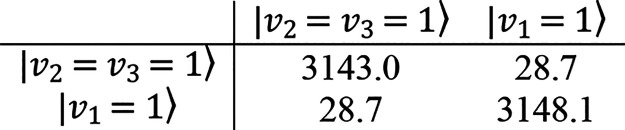
with eigenvalues 3116 and 3173 cm^–1^, and the perturbed levels are almost a 1:1 mixture of the unperturbed ones. In addition, the region presents a large number of weaker features mainly due to two quanta combinations bands, as detailed in Table 11. The last part of the MIR spectrum, in the range of 3500–5000 cm^–1^, shows a number of weak absorption features attributable to binary and ternary combination vibrations with an intensity within 0.5 km mol^–1^. Among these, the most important ones come from the first overtone of ν_2_ (3560 cm^–1^) and the binary combinations involving the ν_1_ vibration, namely, ν_1_ + ν_7_ (3784.9 cm^–1^), ν_1_ + ν_6_ (4087.9 cm^–1^), ν_1_ + ν_5_ (4330.5 cm^–1^), ν_1_ + ν_4_ (4419.6 cm^–1^), ν_1_ + ν_3_ (4523.7 cm^–1^), and ν_1_ + ν_2_ (4951.0 cm^–1^). These combination levels of the type *v*_1_ = *v*_j_ = 1 are expected to inherit the Fermi resonance ν_1_/ν_2_ + ν_3_ and, hence, to be involved to various extents in 1–2 interactions with the corresponding three quanta combination levels *v*_2_ = *v*_3_ = *v*_j_ = 1 that in the present investigation have been treated by manually setting up the proper interaction matrices on the basis of the computed quantities, resulting in an optimal agreement with the experiment. In particular for *v*_2_ = *v*_3_ = *v*_5_ = 1, the resonant systems involving ν_1_/ν_2_ + ν_3_ and ν_5_/ν_6_ + ν_9_ intersect, giving rise to a four-level system that using the spectroscopic parameters evaluated from the ChS:rDSD hybrid force field has been described by the following interaction Hamiltonian:

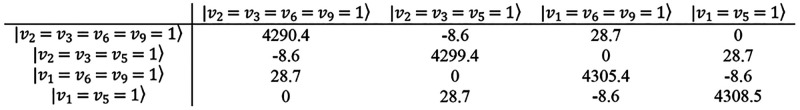



Upon diagonalization, the following eigenvalues and eigenvectors are obtained:

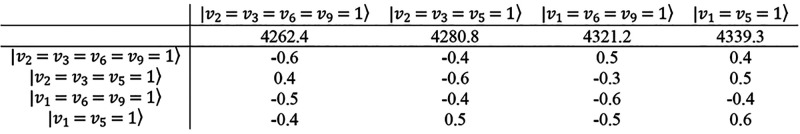



From these, it can be observed that the perturbed levels are better described as a superposition of the unperturbed ones. That said, when the labels corresponding to the most important component are retained, it can be concluded that the four quanta combination is predicted at 4262 cm^–1^, the ν_2_ + ν_3_ + ν_5_ and ν_1_ + ν_6_ + ν_9_ bands move to 4281 and 4321 cm^–1^, respectively, and the ν_1_ + ν_5_ combination is expected around 4339 cm^–1^. Among these, only the ν_2_ + ν_3_ + ν_5_ and ν_1_ + ν_5_ transitions are expected to produce weak but observable absorptions, and indeed, the perturbed transition frequencies well reconcile with the bands observed at 4280.8 and 4330.5 cm^–1^. In passing, it should be noted that the band here assigned as 2ν_2_ was originally attributed to a three quanta combination in ref (43); however, according to the guidance of quantum chemical calculations, the overtone presents the more suited choice with the strongest transition expected at around 3560 cm^–1^.

### 4.4 Absorption Cross Sections, Anharmonic Dipole Moment Surface, and Global Warming Potential

**Figure 4:**
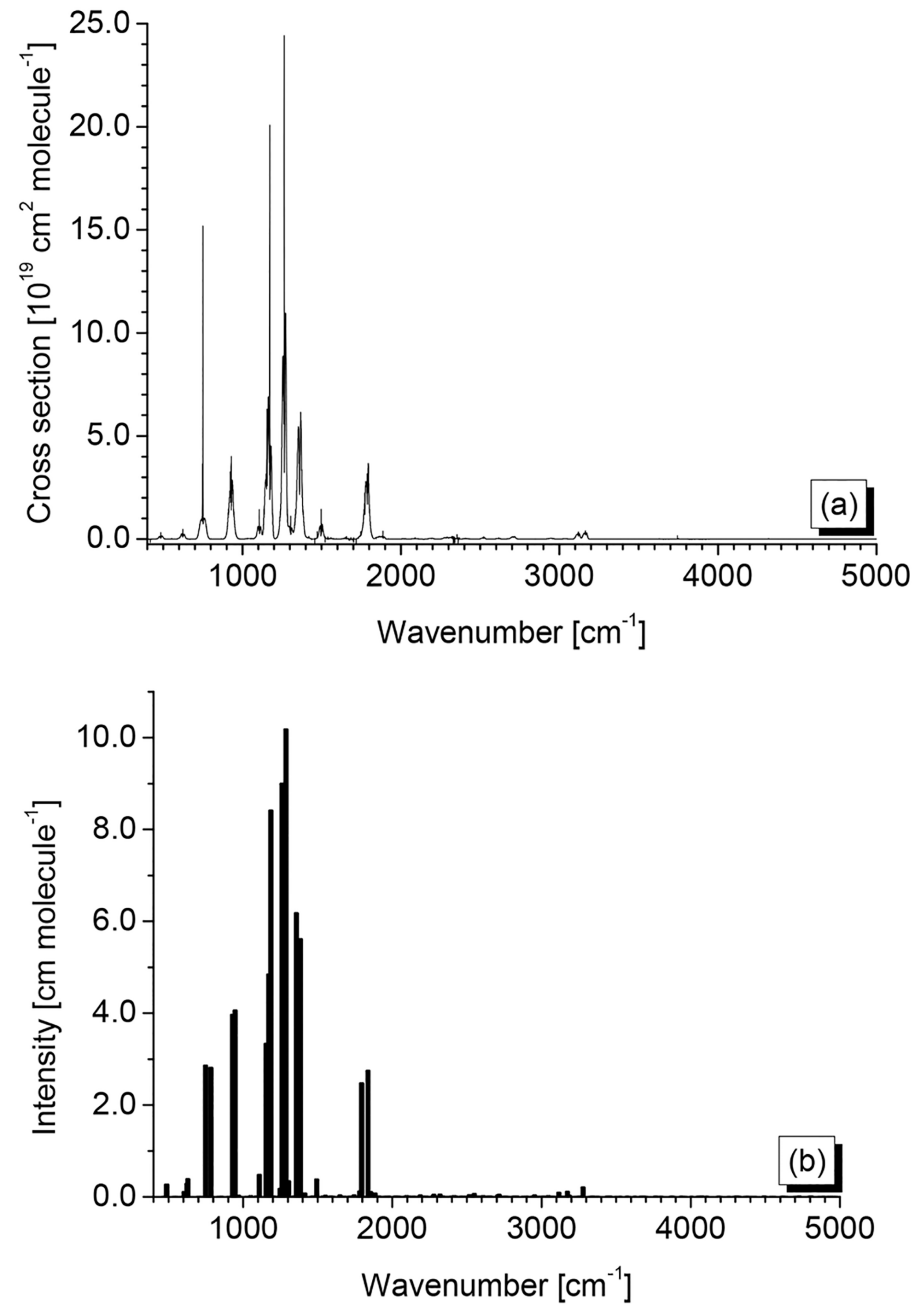
(a) Experimental cross-section spectrum of HFO-1123 in the 400–5000 cm^–1^ spectral region. (b) Theoretical stick spectrum obtained at the hybrid ChS:rDSD level of theory over the same spectral range.

The experimental investigation has also involved the determination of integrated absorption cross sections (i.e., IR band intensities) in the 400–5000 cm^–1^ region. While the reader is referred to the available literature for a detailed description of the procedure,^21,24,34^ here, only the main steps are summarized. The method is based on the least-squares fitting the point-by-point absorbance value, *A*(*ṽ*), measured at each wavenumber, *ṽ*, against the sample concentration. The slope of the straight line so obtained at each wavenumber, σ(*ṽ*), provides the absorbance cross section per molecule (cm^2^ molecule^–1^) with the statistical uncertainty obtained in a similar way. In passing, it should be noted that the procedure yields IR intensities in very good agreement with those determined by the line-by-line analysis of high-resolution spectra (see, for example, refs (51, 85, and 86)). The integrated absorption cross sections obtained from the analysis of the medium resolution IR spectra of HFO-1123 are collected in Table 12, together with the corresponding theoretical predictions at the ChS:rDSD level of theory, while the cross section spectrum is detailed in Figure 4.

**Table 12:** Experimental and Theoretical Integrated Absorption Cross Sections (km mol^–1^) of HFO-1123

spectral range (cm^–1^)	main absorption	exp.^a^	ChS:rDSD^b^
440–530	ν_8_	2.1(1)	2.07
530–570	ν_9_ + ν_12_, ν_11_	0.068(3)	0.01
570–670	ν_7_	3.7(2)	3.81
670–820	ν_10_, ν_8_ + ν_12_	27(2)	29.66
820–1000	ν_6_, 2ν_8_	52(1)	54.96
1000–1205	ν_8_ + ν_11_, ν_10_ + ν_12_, 2ν_11_, ν_6_ + ν_9_, ν_5_, ν_7_ + ν_11_	129(3)	142.37
1205–1445	ν_4_, ν_10_ + ν_11_, ν_3_, ν_6_ + ν_8_	279(3)	313.55
1705–1950	ν_4_ + ν_8_, ν_2_, ν_5_ + ν_7_, ν_3_ + ν_8_, 2ν_6_, ν_4_ + ν_7_, ν_5_ + ν_10_	63(2)	68.97
1950–2240	ν_3_ + ν_7_, ν_2_ + ν_9_, ν_2_ + ν_12_, ν_4_ + ν_6_	1.92(8)	1.88
2460–2640	2ν_4_, ν_3_ + ν_4_	1.62(2)	2.03
2640–2800	ν_2_ + ν_6_	2.24(2)	2.79
2985–3250	ν_2_ + ν_4_, ν_2_ + ν_3_, ν_1_	8.5(3)	11.18
3415–3580	2ν_2_	0.34(4)	0.33
3980–4130	ν_1_ + ν_6_	0.34(3)	0.33
4150–4665	ν_1_ + ν_5_, ν_1_ + ν_4_, ν_1_ + ν_3_	1.08(3)	1.15
4665–5000	ν_1_ + ν_2_	0.23(2)	0.20
MAE^c^			3.97

a Values in parentheses are standard errors in the units of the last significant digits.

b Harmonic frequencies and intensities from ChS, anharmonic contributions at the rev-DSD-PBEP86-D3/jun-cc-pVTZ level.

c Mean absolute error.

As to the greenhouse effect exerted by this molecule, the most important spectral regions are, as expected, those featuring the strong absorptions due to the C–F stretching vibrations. In particular, the spectral region between 1000 and 1445 cm^–1^ accounts for almost 50% of the total IR absorption strength measured over the 400–5000 cm^–1^ range. As previously pointed out, it hosts the absorptions stemming from the ν_3_ and ν_4_ normal modes involving the asymmetric stretching of the CF_2_ moiety and predicted with an anharmonic intensity of 119.7 and 196.4 km mol^–1^, respectively. Next, the C–F stretching motion of the CHF group (ν_5_) is located within the 1000–1205 cm^–1^ spectral range within the atmospheric window, which accounts for about 23% of the overall integrated band intensity. In particular, according to ChS:rDSD anharmonic computations, it has an intensity of 75.5 km mol^–1^, almost half with respect to the harmonic value (140.4 km mol^–1^). The reason for this weakening accounting for both mechanical and electrical anharmonicity has to be sought in the aforementioned Fermi type II interaction with ν_6_ + ν_9_ that borrows intensity from the fundamental, and indeed, its strength is predicted to be 57.9 km mol^–1^. The ranges 820–1000 and 1705–1950 cm^–1^ each contain about the 10% of the total measured intensity. The former, still within the atmospheric window, presents ν_6_, the symmetric CF_2_ stretching, as the only relevant contribution, while the latter is dominated by the ν_2_ C=C stretching vibration whose IR intensity is predicted to be 59.3 km mol^–1^. Then, the spectral intervals 670–820 cm^–1^ (27 km mol^–1^) and 2985–3250 cm^–1^ (8.5 km mol^–1^) contribute to about 5% and 1% of the overall integrated band intensity, respectively. Their measured intensities are mainly due to the ν_10_ vibration, whose computed intensity is 29 km mol^–1^ and the ν_1_ and ν_2_ + ν_3_ bands. In the latter case, as well, the combination vibration steals intensity from the C–H stretching vibration, thus reaching an absorption cross section of about 4 km mol^–1^, while the ν_1_ fundamental loses about 50% of its strength (from 11.4 to 6.3 km mol^–1^) upon introduction of anharmonic effects. The remaining spectral portions account for less than 1% of the total integrated absorption cross section measured over the whole 400–5000 cm^–1^ range. Overall, a good accord between the measured intensities and the corresponding theoretical counterparts is noted for the different integration ranges, the mean absolute error of around 4 km mol^–1^ being coherent with the expected accuracy.^63−65^

Finally, when one utilizes the measured cross section spectrum and the narrowband model of ref (87), the HFO-1123 radiative forcing (RF) is estimated to be 0.10 W m^–2^ ppbv^–1^. Since this approach might not be well suited for short-lived molecules like HFO-1123, the value determined should be taken as an estimate of the actual RF, although the data computed in this way are in good agreement with those obtained using a more sophisticated model on a similar halogenated olefin.^88^ To the best of our knowledge, there is no data about the atmospheric lifetime of HFO-1123, which however is required in order to evaluate the global warming potential index. On the basis of the available literature,^89^ an atmospheric lifetime of 10–30 days appears as a reasonable upper limit guess, leading to an estimated GWP between 2.0 and 5.3 (taking CO_2_ as the reference) on a time horizon of 100 years.

## 5 Conclusions

Several research efforts have been devoted to the study of the infrared spectroscopic behavior of both HFCs and HCFCs with the aim to assess the environmental impacts of these compounds. Indeed, it is important for radiative transfer calculations and atmospherical models to include data about their absorptions, and furthermore, accurate characterizations of their spectral properties constitute the basic requirement for monitoring their concentration in the atmosphere or work places. The present work aimed at investigating the structural and rotational/vibrational spectroscopic behavior of HFO-1123, one of the next generation refrigerants proposed as a replacement for HFCs, which has a supposedly shorter atmospheric lifetime and hence a lower impact on global warming. In particular, an accurate equilibrium geometry has been first worked out by applying post-Hartree–Fock composite methods. On the one side, this has been the first step toward the detailed modeling of its spectroscopic features, particularly those of relevance for rotational spectroscopy. On the other side, it represents the most accurate equilibrium geometry presented up to date for this species. The accuracy reached in the geometry has been mirrored in the accuracy in the computed ground state- and excited fundamental vibrational level- rotational constants of HFO-1123, which showed a remarkable agreement with the available experimental data with deviations well within 0.1%. Quartic and sextic centrifugal distortion parameters have been evaluated as well, addressing the dichotomy existing on the experimental data determined for the latter terms. Together with the α_k_^β^ vibration–rotation constants and the ζ_kl_^a^ Coriolis coupling terms, the computed data can be used to drive further high-resolution vibrational–rotational investigations. Attention has then been moved to the integrated experimental–theoretical investigation of HFO-1123 vibrational spectra. From the theoretical point of view, accurate harmonic frequencies and IR intensities have been obtained using the ChS method, and subsequently, these have been combined with anharmonic contributions evaluated using the rev-DSDPBEP86 functional that has been shown to provide excellent performances for the computation of vibrational transition frequencies and intensities. The anharmonic spectra simulated in this way have been used to assist the characterization of the experimental gas-phase spectra. To this end, the observed absorption features have been identified as fundamentals, overtones, a combination, and hot bands up to three quanta of vibrational excitations, and the analysis resulted in the assignment of almost 120 vibrational transitions in the 200–5000 cm^–1^ spectral range. A number of 1–2 Fermi resonances have been recognized and consistently treated, thus explaining the observed spectral pattern and intensity alterations. Furthermore, the integrated absorption cross sections have been experimentally determined for the spectral region between 400 and 5000 cm^–1^, reporting also in this case a very good agreement with the theoretical predictions that have furnished a quantitative description of the overall band shape over the whole spectral region investigated in the present work. Finally, the obtained spectroscopic data have been employed to obtain an estimate of the HFO-1123 radiative forcing. From this, an upper limit of the global warming potential has been determined, which is of interest in view of the use of HFO-1123 for refrigeration applications.
